# Supplementary material for: Multi‐dataset identification of innovative feature genes and molecular mechanisms in keratoconus
Source: J Cell Mol Med. 2024 Sep 19;28(18):e70079. doi: 10.1111/jcmm.70079 (PMC11412914; doi:10.1111/jcmm.70079)
Supplement: Supplementary file 2 — Material S2. [file JCMM-28-e70079-s002.zip › 7.GSEA analysis/3.DBNDD1/KEGG_MELANOGENESIS.html]

Details for gene set KEGG\_MELANOGENESIS[GSEA]

|  || Dataset | DBNDD1.group.cls#h\_versus\_l |
| Phenotype | group.cls#h\_versus\_l |
| Upregulated in class | l |
| GeneSet | KEGG\_MELANOGENESIS |
| Enrichment Score (ES) | -0.3780803 |
| Normalized Enrichment Score (NES) | -1.419139 |
| Nominal p-value | 0.06483301 |
| FDR q-value | 0.68674767 |
| FWER p-Value | 0.881 |
Table: GSEA Results Summary

  

Fig 1: Enrichment plot: KEGG\_MELANOGENESIS      
 Profile of the Running ES Score & Positions of GeneSet Members on the Rank Ordered List

  

| SYMBOL | TITLE | RANK IN GENE LIST | RANK METRIC SCORE | RUNNING ES | CORE ENRICHMENT || 1 | CAMK2B | na | 217 | 0.536 | 0.0413 | No |
| 2 | MC1R | na | 2945 | 0.254 | -0.0149 | No |
| 3 | CAMK2A | na | 3995 | 0.213 | -0.0262 | No |
| 4 | CALML6 | na | 4335 | 0.202 | -0.0181 | No |
| 5 | PRKCG | na | 6020 | 0.150 | -0.0534 | No |
| 6 | ADCY7 | na | 6033 | 0.150 | -0.0404 | No |
| 7 | ADCY9 | na | 6468 | 0.137 | -0.0408 | No |
| 8 | ADCY6 | na | 6632 | 0.133 | -0.0336 | No |
| 9 | WNT16 | na | 6810 | 0.129 | -0.0273 | No |
| 10 | CALML5 | na | 7930 | 0.105 | -0.0503 | No |
| 11 | ADCY4 | na | 8335 | 0.097 | -0.0533 | No |
| 12 | ADCY5 | na | 8470 | 0.095 | -0.0487 | No |
| 13 | PLCB2 | na | 9009 | 0.086 | -0.0566 | No |
| 14 | CREBBP | na | 9242 | 0.082 | -0.0561 | No |
| 15 | FZD5 | na | 9252 | 0.082 | -0.0491 | No |
| 16 | FZD3 | na | 9718 | 0.075 | -0.0558 | No |
| 17 | FZD4 | na | 9743 | 0.075 | -0.0498 | No |
| 18 | MAPK3 | na | 9865 | 0.073 | -0.0468 | No |
| 19 | WNT9A | na | 9937 | 0.072 | -0.0425 | No |
| 20 | CALML3 | na | 10231 | 0.068 | -0.0449 | No |
| 21 | FZD1 | na | 10494 | 0.065 | -0.0467 | No |
| 22 | WNT8A | na | 10620 | 0.063 | -0.0447 | No |
| 23 | MAP2K2 | na | 10931 | 0.059 | -0.0484 | No |
| 24 | CALM2 | na | 11915 | 0.048 | -0.0726 | No |
| 25 | ASIP | na | 12173 | 0.045 | -0.0760 | No |
| 26 | CREB3 | na | 12277 | 0.045 | -0.0750 | No |
| 27 | FZD8 | na | 13456 | 0.035 | -0.1059 | No |
| 28 | CAMK2G | na | 13529 | 0.034 | -0.1050 | No |
| 29 | GNAO1 | na | 13702 | 0.032 | -0.1071 | No |
| 30 | WNT10B | na | 14038 | 0.030 | -0.1141 | No |
| 31 | KRAS | na | 14127 | 0.029 | -0.1141 | No |
| 32 | GNAI3 | na | 14246 | 0.028 | -0.1150 | No |
| 33 | WNT3A | na | 14444 | 0.027 | -0.1183 | No |
| 34 | FZD6 | na | 14642 | 0.025 | -0.1217 | No |
| 35 | GNAI2 | na | 15169 | 0.022 | -0.1350 | No |
| 36 | CREB3L1 | na | 15797 | 0.017 | -0.1516 | No |
| 37 | PRKX | na | 15799 | 0.017 | -0.1501 | No |
| 38 | EDNRB | na | 15833 | 0.017 | -0.1495 | No |
| 39 | CALM1 | na | 15870 | 0.017 | -0.1491 | No |
| 40 | CTNNB1 | na | 16378 | 0.013 | -0.1625 | No |
| 41 | WNT5A | na | 17212 | 0.008 | -0.1859 | No |
| 42 | POMC | na | 18926 | -0.004 | -0.2350 | No |
| 43 | HRAS | na | 18984 | -0.004 | -0.2362 | No |
| 44 | RAF1 | na | 19005 | -0.005 | -0.2364 | No |
| 45 | ADCY2 | na | 19167 | -0.006 | -0.2405 | No |
| 46 | KIT | na | 19563 | -0.009 | -0.2511 | No |
| 47 | PLCB3 | na | 19605 | -0.010 | -0.2515 | No |
| 48 | PLCB4 | na | 19707 | -0.010 | -0.2535 | No |
| 49 | TCF7L2 | na | 20073 | -0.014 | -0.2628 | No |
| 50 | DVL3 | na | 20205 | -0.015 | -0.2653 | No |
| 51 | WNT5B | na | 20321 | -0.016 | -0.2672 | No |
| 52 | CREB3L3 | na | 20550 | -0.018 | -0.2722 | No |
| 53 | CALM3 | na | 20684 | -0.019 | -0.2743 | No |
| 54 | PRKACA | na | 20814 | -0.020 | -0.2763 | No |
| 55 | PRKCA | na | 21412 | -0.026 | -0.2912 | No |
| 56 | WNT4 | na | 21797 | -0.030 | -0.2996 | No |
| 57 | GNAQ | na | 21825 | -0.030 | -0.2977 | No |
| 58 | WNT3 | na | 21833 | -0.030 | -0.2952 | No |
| 59 | GNAS | na | 22042 | -0.033 | -0.2983 | No |
| 60 | GSK3B | na | 22452 | -0.037 | -0.3069 | No |
| 61 | CREB3L4 | na | 22477 | -0.037 | -0.3043 | No |
| 62 | KITLG | na | 22603 | -0.039 | -0.3045 | No |
| 63 | MAPK1 | na | 22705 | -0.040 | -0.3039 | No |
| 64 | WNT2 | na | 22923 | -0.042 | -0.3064 | No |
| 65 | ADCY8 | na | 23069 | -0.044 | -0.3067 | No |
| 66 | ADCY3 | na | 23170 | -0.045 | -0.3055 | No |
| 67 | WNT9B | na | 23327 | -0.047 | -0.3058 | No |
| 68 | TCF7 | na | 23571 | -0.051 | -0.3084 | No |
| 69 | CREB3L2 | na | 23589 | -0.051 | -0.3043 | No |
| 70 | DVL1 | na | 24203 | -0.059 | -0.3168 | No |
| 71 | EP300 | na | 24292 | -0.060 | -0.3140 | No |
| 72 | CREB1 | na | 24296 | -0.060 | -0.3087 | No |
| 73 | WNT6 | na | 24905 | -0.069 | -0.3201 | No |
| 74 | DVL2 | na | 25071 | -0.072 | -0.3185 | No |
| 75 | FZD9 | na | 25389 | -0.077 | -0.3208 | No |
| 76 | GNAI1 | na | 25682 | -0.082 | -0.3220 | No |
| 77 | DCT | na | 26024 | -0.088 | -0.3241 | No |
| 78 | WNT7B | na | 26237 | -0.092 | -0.3220 | No |
| 79 | PRKACB | na | 26730 | -0.102 | -0.3271 | No |
| 80 | TCF7L1 | na | 26813 | -0.104 | -0.3203 | No |
| 81 | NRAS | na | 27014 | -0.108 | -0.3165 | No |
| 82 | WNT10A | na | 27282 | -0.114 | -0.3140 | No |
| 83 | PRKACG | na | 28125 | -0.134 | -0.3264 | No |
| 84 | MAP2K1 | na | 29246 | -0.164 | -0.3442 | No |
| 85 | ADCY1 | na | 29881 | -0.185 | -0.3461 | No |
| 86 | EDN1 | na | 30990 | -0.220 | -0.3585 | Yes |
| 87 | WNT8B | na | 31267 | -0.231 | -0.3459 | Yes |
| 88 | FZD10 | na | 31652 | -0.246 | -0.3351 | Yes |
| 89 | WNT7A | na | 31957 | -0.261 | -0.3207 | Yes |
| 90 | PLCB1 | na | 32023 | -0.265 | -0.2990 | Yes |
| 91 | FZD2 | na | 32387 | -0.287 | -0.2840 | Yes |
| 92 | TYRP1 | na | 32829 | -0.316 | -0.2687 | Yes |
| 93 | LEF1 | na | 32857 | -0.317 | -0.2413 | Yes |
| 94 | CAMK2D | na | 32964 | -0.326 | -0.2154 | Yes |
| 95 | WNT2B | na | 33588 | -0.378 | -0.1998 | Yes |
| 96 | TYR | na | 33779 | -0.401 | -0.1696 | Yes |
| 97 | WNT1 | na | 33810 | -0.404 | -0.1346 | Yes |
| 98 | FZD7 | na | 33835 | -0.408 | -0.0990 | Yes |
| 99 | WNT11 | na | 33881 | -0.415 | -0.0635 | Yes |
| 100 | PRKCB | na | 34153 | -0.452 | -0.0311 | Yes |
| 101 | MITF | na | 34462 | -0.537 | 0.0077 | Yes |
Table: GSEA details [plain text format]

  

Fig 2: KEGG\_MELANOGENESIS      
 Blue-Pink O' Gram in the Space of the Analyzed GeneSet

  

Fig 3: KEGG\_MELANOGENESIS: Random ES distribution      
 Gene set null distribution of ES for **KEGG\_MELANOGENESIS**
